# Draft genome sequence of *Bacillus paranthracis* S23, isolated from saline soil of Lake Bogoria, Kenya

**DOI:** 10.1128/mra.00893-26

**Published:** 2026-09-18

**Authors:** Tofick Barasa Wekesa, Grace Kennedy, Damaris Barminga, Alena Petra Wekesa, Martin Ng'ang'a Muigano, Mercy Achieng Wasonga, Ndinda Kavesu, Zipporah Muchiri, Samuel Mwakisha Mwamburi, Christine Migwi

**Affiliations:** 1 Institute of Biotechnology Research, Jomo Kenyatta University of Agriculture and Technology 118985, Nairobi, Kenya; 2 Department of Organismal Biology, Uppsala University 220035, Uppsala, Sweden; 3 Ecobalance Biocontrol Limited, Nairobi, Kenya; 4 Bio One Scientific, Nairobi, Kenya; 5 Embu University 472187, Embu, Kenya; 6 TerraVerge Solutions Limited, Mombasa, Kenya; 7 Kenya Marine and Fisheries Research Institute 117470, Mombasa, Kenya; University of Manitoba, Winnipeg, Canada

**Keywords:** *Bacillus paranthracis*, draft genome, Mastitis, Lake Bogoria

## Abstract

*Bacillus paranthracis* S23 was isolated from saline soil of Lake Bogoria. Its draft genome, generated using Illumina HiSeq sequencing, comprises 5,583,771 bp across 205 contigs and contains 5,741 predicted protein-coding genes. Genome mining identified biosynthetic gene clusters associated with the production of secondary metabolites, such as bacillibactin and fengycin-like compounds.

## ANNOUNCEMENT

*Bacillus paranthracis* is a gram-positive, rod-shaped, endospore-forming bacterium within the *Bacillus cereus* group (1). It has been reported from diverse environments, including soil, rhizosphere, and aquatic habitats (2). Previous studies have described potentially effect of *B. paranthracis* strains causing bovine mastitis. However, *B. paranthracis* strain MHSD3, isolated as a bacterial endophyte, possesses antimicrobial secondary metabolites and biosynthetic genes with antimicrobial compounds (3). *B. paranthracis* strain Endo10 is reported to promote tomato growth and enhance plant tolerance to salinity stress (2). The biological properties of individual *B. paranthracis* strains may differ, and the specific activities of strain S23 have not previously been characterized at the genomic level.

*Bacillus paranthracis* strain S23 was isolated from a soil sample collected at Lake Bogoria, Kenya (N 10°43.692′, E 058°25.621′), as part of a previously reported survey of culturable bacteria (4). Briefly, soil samples were collected aseptically and processed by serial dilution (5), followed by spread plating (6) on modified nutrient agar (HiMedia, India). Plates were incubated at 35°C for 48 h, and distinct colonies were purified by repeated streaking.

Genomic DNA was extracted using the cetyltrimethylammonium bromide protocol described by Liu et al. (7). High-quality genomic DNA was submitted to Inqaba Biotec (South Africa) for library preparation using the Illumina DNA Prep Kit (Illumina, USA) according to the manufacturer’s instructions, followed by whole-genome sequencing on an Illumina HiSeq platform using 2 × 150 bp paired-end reads.

Raw sequencing reads were assessed using FastQC v.0.12.1 (8) before quality filtering and adapter removal with Fastp v.1.0.1 (9). Filtered reads were assembled *de novo* using SPAdes v.4.2.0 (10) with default parameters, and assembly quality was evaluated using QUAST v.5.0.2 (11).

A total of 2,906,508 paired-end Illumina reads were generated, corresponding to an estimated sequencing depth of 78×. The assembled draft genome of *Bacillus paranthracis* S23 comprises 205 contigs totaling 5,583,771 bp, with a GC content of 35.23% and an N50 of 75,314 bp. Genome annotation using Prokka v.1.2.0 (12) predicted 5,741 genes, including 10 rRNA genes, 68 tRNA genes, and 1 tmRNA gene. Genome completeness was estimated at 99.41%, with 0.29% contamination and 0.00% strain heterogeneity using CheckM v.1.1.3 (13) based on a *Bacillus*-specific marker set.

Genome mining using antiSMASH v.8.0.2 (14) identified 11 putative secondary metabolite biosynthetic gene clusters, including NRPS, RiPP-like, LAP, terpene, and β-lactone pathways. These included biosynthetic gene clusters showing similarity to bacillibactin, fengycin, and a molybdenum cofactor-associated terpene cluster (15). Several additional RiPP-like and NRPS clusters displayed little or no similarity to known biosynthetic pathways, suggesting that strain S23 may encode previously undescribed natural products (16).

The predicted fengycin-like and bacillibactin biosynthetic clusters suggest potential roles in secondary metabolite production and iron acquisition, respectively, warranting further experimental investigation (2, 17).

We anticipate that the genome sequence of *B. paranthracis* S23 will facilitate investigations into secondary metabolite biosynthesis, adaptation to saline and alkaline environments, and the functional characterization of its potential antimicrobial and bovine mastitis.

## Data Availability

The Whole Genome Shotgun project of *Bacillus paranthracis* strain S23 has been deposited in the DDBJ/ENA/GenBank databases under BioProject PRJNA1493057, BioSample SAMN61547497, and SRA SRR39797221.
